# Family involvement practices for persons with psychotic disorders in community mental health centres – a cross-sectional fidelity-based study

**DOI:** 10.1186/s12888-021-03300-4

**Published:** 2021-06-02

**Authors:** Lars Hestmark, Kristin Sverdvik Heiervang, Reidar Pedersen, Kristiane Myckland Hansson, Torleif Ruud, Maria Romøren

**Affiliations:** 1 Centre for Medical Ethics, University of Oslo, Kirkeveien 166 Fredrik Holsts hus, 0450 Oslo, Norway; 2 Division of Mental Health Services, Akershus University Hospital, Sykehusveien 25, 1474 Nordbyhagen, Norway; 3 Institute of Clinical Medicine, University of Oslo, Oslo, Norway

**Keywords:** Family involvement, Psychotic disorders, Mental health services research, Schizophrenia, Family psychoeducation, Fidelity scale, Implementation science

## Abstract

**Background:**

Family involvement for persons with psychotic disorders is supported by scientific evidence, as well as legal and ethical considerations, and recommended in clinical practice guidelines. This article reports a cross-sectional measurement of the level of implementation of such guidelines in fifteen community mental health centre units in Norway, and presents a novel fidelity scale to measure basic family involvement and support. The aim was to investigate current family involvement practices comprehensively, as a basis for targeted quality improvement.

**Methods:**

We employed three fidelity scales, with 12–14 items, to measure family involvement practices. Items were scored from 1 to 5, where 1 equals no implementation and 5 equals full implementation. Data was analysed using descriptive statistics, a non-parametric test, and calculation of interrater reliability for the scales.

**Results:**

The mean score was 2.33 on the fidelity scale measuring basic family involvement and support. Among patients with psychotic disorders, only 4% had received family psychoeducation. On the family psychoeducation fidelity assessment scale, measuring practice and content, the mean score was 2.78. Among the eight units who offered family psychoeducation, it was 4.34. On the general organizational index scale, measuring the organisation and implementation of family psychoeducation, the mean score was 1.78. Among the units who offered family psychoeducation, it was 2.46. As a measure of interrater reliability, the intra-class correlation coefficient was 0.99 for the basic family involvement and support scale, 0.93 for the family psychoeducation fidelity assessment scale and 0.96 for the general organizational index scale.

**Conclusions:**

The implementation level of the national guidelines on family involvement for persons with psychotic disorders was generally poor. The quality of family psychoeducation was high, but few patients had received this evidence-based treatment. Our novel fidelity scale shows promising psychometric properties and may prove a useful tool to improve the quality of health services. There is a need to increase the implementation of family involvement practices in Norway, to reach a larger percentage of patients and relatives.

**Trial registration:**

ClinicalTrials.gov Identifier NCT03869177. Registered 11.03.19.

**Supplementary Information:**

The online version contains supplementary material available at 10.1186/s12888-021-03300-4.

## Background

Family involvement practices in adult mental health services vary considerably, in terms of both quantity and quality. In this study, we focus on practices towards patients with psychotic disorders [1] and their relatives, but our methods and findings may be relevant to all health services dealing with severe and chronic illness.

Previous research describes how relatives of patients with severe mental illness report a lack of adequate information, support, and cooperation from mental health services [2, 3]. Studies have also documented the overall poor implementation of standardised family interventions in mental health care [4, 5], despite evidence of beneficial outcomes for both patients and relatives [6–15]. Family psychoeducation (FPE) is one such structured family intervention and a cornerstone of the evidence-based treatment of psychotic disorders. It begins with separate alliance sessions with patient and relative(s) and continues with joint psychoeducative sessions, communication skills exercises, and problem-solving sessions [16]. Mental health services may lack the capacity to offer such labour-intensive interventions to all of their eligible patients, and in a few cases the intervention is unnecessary or even contraindicated. Lack of resources, training and capacity are examples of organisational barriers to family involvement, but there are also significant clinical barriers, related to the perspectives of professionals, families, and patients [4, 17, 18].

However, clinicians should always attempt to establish a connection with the patient’s relatives to assess their resources, burdens and needs as informal carers, and listen to their experiences, concerns and preferences. Relatives should be encouraged to provide information concerning the patient and should receive general information on the illness, the health services, and where to obtain further support if necessary. This basic level of family involvement and support is important in all cases of chronic and severe illness. In adult mental health services, it may be viewed as the obligatory basis of a pyramid that extends further to include family psychoeducation, consultation, and family therapy, depending on the families’ needs [19].

In Norway, the Directorate of Health has provided general recommendations on family involvement and support in the health- and care services [20]. These are based on discussions between key stakeholders and experts, research evidence, and ethical considerations. They also include the legal regulations, whereby the health- and care services are obligated to provide information, guidance, support, and appropriate involvement to relatives, who have a corresponding right to these services. The Directorate has also issued specific guidelines on the treatment of psychotic disorders, recommending standardised family interventions as a first-line treatment during all phases of the illness [21], in line with comparable guidelines in other countries [22–25]. In this article, we refer to the general and specific guidelines on family involvement collectively as ‘the national guidelines’.

To improve family involvement practices for persons with psychotic disorders, we needed a systematic and comprehensive assessment of the current level of implementation of these national guidelines. Our hypothesis was that the level of implementation would be low. As part of the ‘Implementation of Family Involvement for persons with Psychotic disorders’ – (IFIP) trial [26], we conducted a baseline assessment of family involvement practices in all fifteen participating community mental health centre (CMHC) units, using three fidelity scales. Measuring program fidelity is an established strategy in implementation science and mental health services research, providing a standardised assessment of evidence-based practices [27]. In order to investigate and implement basic family involvement and support, we developed a novel fidelity scale. The purpose of this article is both to present this new fidelity scale and to report the results from the baseline assessment. To our knowledge, this paper describes the first systematic, comprehensive and broad investigation of family involvement practices in CMHCs to date.

## Methods

This article conforms to the ‘Strengthening the reporting of observational studies in epidemiology (STROBE) statement’ [28] (Additional file 1).

### Study design, setting and participating sites

The investigation reported here is a cross-sectional sub-study of the IFIP trial. The trial as a whole employs a cluster randomised controlled design, where a cluster is defined as one or more CMHC outpatient units with the main responsibility for treating patients with psychotic disorders in their discreet catchment area. We accepted all such units and invited all the sixteen CMHCs in five counties of the South-Eastern Norway Regional Health Authority to participate in the trial. Fifteen clinical sites from twelve CMHCs agreed to participate, including both rural and urban units. The main reason given for non-participation among the remaining CMHCs was a lack of capacity to take part in a research project. Two of the participating sites were merged into one cluster, but were scored separately in this baseline assessment, which took place before randomisation. After randomisation, half of the clusters will receive training and support for 18 months to implement recommendations from the national guidelines, whereas the other half will be given training and support after the 18 months period. The sample size was calculated for the IFIP trial as a whole [26]. Table 1 sums up the characteristics of the clinical sites, and demonstrates the variation in organisation and structure among the services offered to patients with psychotic disorders in Norway.

**Table 1:** Description of the 15 clinical sites (in sequence according to catchment area population)

Site	Catchment area population	Type of unit^a^	Full-time equivalent staff (FTE)	Total number of patients	Patients per FTE	Number of patients with psychotic disorders (F20–29)	Type of patients with F20–29^b^	Patients with F20–29 on community treatment order (CTO)
1	26.000	GPC, AOT	23.1	903	39.1	63	Early, Long-term	11 (17.5%)
2	29.000	AOT	6.3	112	17.8	30	Long-term	10 (33.3%)
3	29.000	AOT	7.5	85	11.3	28	Long-term	3 (10.7%)
4	36.000	DDT	9.8	188	19.2	57	Long-term	15 (26.3%)
5	45.000	AOT	1	28	28	28	Long-term	6 (21.4%)
6	58.000	GPC, AOT, POC	33.35	678	20.3	86	Early, Long-term	32 (37.2%)
7	63.000	AOT	12	175	14.6	100	Long-term	20 (20%)
8	66.000	AOT, POC	10	154	15.4	54	Early, Long-term	8 (14.8%)
9	111.000	POC	12	88	7.3	76	Early, Long-term	19 (25%)
10	118.000	AOT	12.1	186	15.4	148	Long-term	22 (14.9%)
11	130.000	POC	15.5	164	10.6	142	Early, Long-term	12 (8.5%)
12	135.000	POC	20	124	6.2	104	Early, Long-term	28 (26.9%)
13	140.000	POC	28	350	12.5	149	Early, Long-term	79 (53%)
14	150.000	POC	30	417	13.9	217	Early, Long-term	36 (16.5%)
15	175.000	POC	21	231	11	110	Early, Long-term	29 (26.4%)
Total	1.311.000	–	241.65	3883	–	1392	–	330 (23.7%)

^a^ *AOT* Assertive outreach team, *DDT* Dual diagnosis team, *POC* Psychosis outpatient clinic, *GPC* General psychiatric clinic

^b^ Early: Patients with newly diagnosed psychotic disorder. Long-term: Patients with chronic psychotic disorder

### Instruments

In the present survey, we employed three fidelity scales with 12–14 items. In each scale, the items are scored from 1 to 5, where 1 equals no implementation and 5 equals full implementation. The item scores are summed up and divided by the number of items in the respective scale, to produce an average score.

#### The BFIS scale

The project group developed a 14-item fidelity scale to measure the structure, content, penetration rate and implementation of Basic Family Involvement and Support (BFIS) (Additional file 2). The purpose of the scale is to operationalise the national recommendations on family involvement and support in the health- and care services. We were unable to find any similar fidelity scale in the published scientific literature.

The study protocol describes how the project group developed the IFIP intervention, by selecting recommendations from the national guidelines and clustering them into the following key elements [26]:

Clinical interventions:

1. A basic level of family involvement and support.
2. Family psychoeducation (FPE) in single-family groups.

Implementation interventions:

1. Training and guidance of health personnel.
2. A family coordinator.
3. Other implementation measures.

In parallel with that process, we identified model dimensions and items for the BFIS scale to cover the key elements of the IFIP intervention, apart from FPE (Table 2). A detailed account of the IFIP intervention is available in the study protocol [26].

**Table 2:** Key elements of the Basic Family Involvement and Support (BFIS) scale

Element	Recommendation/purpose	Items	Key references
*Structural/ implementation measures*	*The unit should:*		
Training and supervision of health personnel	Provide all clinicians with basic competence and skills, to ensure that family involvement becomes one of the cornerstones of treatment, rather than optional or random.	1	[20, 29]
Family coordinator – General structure and responsibilities	Appoint a family coordinator to help implement and sustain the practice. Relevant tasks may include writing and/or updating written material, arranging information courses for relatives, providing tools, internal training and supervision to local personnel, overseeing implementation efforts and being part of the implementation team.	2	[20]
Implementation measures	Establish an implementation team to organise and supervise the implementation process, and ensure management commitment.	14	[29]
*Routines/procedural measures*			
Identification and documentation of the relatives	Ensure that personnel identify and document the relatives. This is fundamental to establish any kind of family involvement. The scale rates only the penetration rate of identifying and documenting the next of kin and children, but it is also helpful to identify other important persons and the extended network.	5	[20]
Documentation of family involvement in the patient’s discharge report	Ensure that clinicians document family involvement in the patient’s discharge report, so that other clinicians in specialist health services or municipalities, who will care for the patient, get an overview of the family involvement conducted so far to establish a continuity of care.	13	[20, 30, 31]
*Clinical measures*			
Conversation(s) with the patient focusing on family involvement	Offer patients with psychotic disorders at least one consultation/conversation, where the major part is dedicated to discuss family involvement and FPE. A way to standardise the content of such conversations is to employ a checklist, with necessary adjustments to the patient’s specific needs.	3,6,7	[6, 20, 21]
Conversation(s) with the relative(s) focusing on family involvement	Offer relatives at least one conversation without the patient present. This provides them with an opportunity to express how the patient’s illness affect their lives, without fearing how this information might affect the patient. These conversations are modelled after the ‘alliance sessions’ in the FPE-model and can be standardised by using a checklist.	4,8	[16, 19, 20]
Conversation(s) with the patient and relative(s) together focusing on family involvement	Offer the patient and relative(s) a conversation together. This could be an introductory conversation to agree on some rules for the separate conversations, or it could be after the separate conversations to sum up the things that can be shared. The conversation might also constitute the initial phase of FPE. The patient’s primary clinician should attend at least one such conversation to assure the integration between family involvement and other treatment strategies.	9,10	[16, 20]
Developing a crisis/coping plan	Ensure that a crisis/coping plan is made, ideally when the patient is competent and/or in a stable phase of the illness. It should be regularly updated and include the patient’s preferences if the illness worsens, and relevant contacts. Relatives may contribute to, or should at least be made familiar with, its contents.	11	[21, 32–34]
Information meetings/ psychoeducative seminars for relatives	Offer relatives information meetings/psychoeducative seminars. This is particularly important for relatives of patients who refuse to participate in FPE, or refuse any contact between health professionals and relative(s).	12	[11, 20]

While developing the BFIS scale, we sought to include items measuring both practice and penetration rate. By ‘penetration rate’, we mean the percentage of eligible patients and/or relatives that receive a certain invitation, treatment, service or practice. Consequently, the scale emphasises the importance of reaching a significant amount of patients and relatives, while also practicing the model accurately. Although the scale rates whether the unit provides annual training in basic family involvement and support (item 1), we did not require such training of the local clinicians in order for the unit to achieve scores on clinical practice items (items 3–13). In addition to clinical elements, the scale measures implementation elements to investigate the organisation and structure of family involvement practices. Thus, we intend the scale to give a comprehensive picture of the status quo, as well as being able to monitor changes during an implementation process.

The project group followed the standardised procedures for scale development described by Bond and colleagues [35]. One exception was that the scale had limited piloting because of time constraints. Some items were therefore eliminated or changed after the baseline data were collected. Where additional data and clarifications were required to adjust the scores, fidelity assessors did follow-up interviews with local personnel by phone. The baseline scores and reports were adjusted a second time after the fidelity assessments at 6 months follow-up, which resulted in some minor changes to the scale. Only the consensus scores were adjusted, in order not to interfere with the calculation of interrater reliability, except where eliminated items resulted in changes to the individual scores.

#### The FPE scale and the GOI scale

The 14-item Family Psychoeducation Fidelity Assessment (FPE) scale was used to measure the practice and content of FPE. This scale has demonstrated acceptable psychometric properties in previous trials, including in a Norwegian translation and context [36, 37]. The 12-item General Organizational Index (GOI) scale provided a complementary assessment of FPE’s integration in the unit’s practice, by measuring individualisation, quality improvement, program philosophy, and penetration rate. A recent study reported acceptable psychometric properties of the GOI scale, when used to assess the implementation of Illness Management and Recovery in Norway [38]. In both scales, an average score of 4 or above indicates adequate implementation, whereas scores below 4 signals low implementation. By convention, the sites that did not offer FPE to patients and their families were scored 1 on all items in the FPE scale and GOI. Item 7: ‘prodromal signs’ in the FPE scale was not scored, since the units in question treated patients with an established or tentative diagnosis of psychotic disorder, rather than prodromal or Ultra High Risk states.

### Data collection

The clinical units were recruited during the three first quarters of 2018, and baseline assessments took place during November and December that same year. Trained fidelity assessors visited each unit and measured fidelity by performing structured interviews with leaders, clinicians and resource-persons, and by reviewing written material such as procedures, checklists, information leaflets, invitation letters, and didactic material. The head of the unit (department-, section- or team leader) was interviewed individually, whereas team leaders (if applicable), clinicians, and resource-persons (if applicable) were interviewed in separate or combined groups of 2–5 persons, with a total of 2–4 interviews of 1–1,5 h length at each site. We also collected organisational data (Table 1).

At each site, the two fidelity assessors first scored all items independently and then reached a consensus score for each item. The assessors, and the pairing of them, varied between sites. They were drawn from a pool of five researchers, who were also health professionals, but none of them worked at the clinical sites in the study. We exclusively assessed the units’ practice towards patients with psychotic disorders and their relatives. The fidelity assessors prepared a detailed report for each site to accompany the scores. Scores and reports were sent to the units in the intervention arm after randomisation, for them to correct any misunderstandings or misconceptions, and to adjust scores if necessary. However, none of the units gave any feedback that resulted in a score adjustment. The sites in the control arm did not receive their scores or reports, to avoid influencing their practice during the intervention period.

### Data analysis

We examined item distributions for all three scales, including means, ranges, standard deviations and number of sites achieving low, adequate and full implementation of the various items. Based on organisational data from the clinical sites, we calculated the percentage of patients with psychotic disorders who had received FPE.

For the BFIS scale, we calculated the percentage of exact agreement for each item. We also investigated interrater reliability (IRR) by calculating the Intra-class Correlation Coefficient (ICC) for total mean fidelity and for each item, using a one-way random effects analysis of variance model for agreement between two assessors. By employing the same model, we calculated the ICC for the FPE scale and the GOI scale. To investigate a possible correlation, between whether the units offered FPE and the BFIS scale scores, we employed an independent samples Mann-Whitney U test. All data analyses were carried out using SPSS version 26.

## Results

### Basic family involvement and support

Item distributions and interrater reliability for the BFIS scale are listed in Table 3. The mean BFIS score in fifteen sites was 2.33, ranging from 1.57 to 3.79. None of the sites had annual training of their health professionals in family involvement and support. Personnel had access to supervision on the subject in eleven of the sites (item 1). Only four sites had health professionals designated to coordinate family involvement and support (item 2). Their responsibilities varied and one of them did not have allocated time to the task. In accordance with the law, all units had procedures and health personnel responsible for taking care of children as next of kin.

**Table 3:** Item distributions and interrater reliability for the Basic Family Involvement and Support (BFIS) scale (*n* = 15)

Item	Description	Mean (SD)	Number of sites achieving score			Agreement (%)	ICC
			1–3	4	5		
	*Structure, content and implementation subscale*						
1	Training and supervision of health personnel	1.00 (0.00)	15	0	0	100	1.000
2	Family coordinator	1.53 (0.99)	14	1	0	87	0.924
3	Conversation(s) with the patient	2.47 (0.64)	14	1	0	80	0.858
4	Conversation(s) with the relative(s)	2.40 (0.91)	14	0	1	60	0.917
14	Implementation measures	1.00 (0.00)	15	0	0	100	1.000
	Subscale total	1.68 (0.43)	15	0	0	–	0.931
	*Penetration rate subscale*						
5	Identifying/ documenting the relatives	5.00 (0.00)	0	0	15	100	1.000
6	Conversation(s) with the patient	1.27 (1.03)	14	0	1	100	1.000
7	Discussing family involvement	4.13 (0.83)	4	5	6	73	0.904
8	Conversation(s) with the relative(s)	1.60 (0.83)	14	1	0	80	0.940
9	Conversation(s) with the patient and relative(s)	2.73 (1.22)	11	3	1	73	0.952
10	Primary clinician attends one meeting	2.60 (1.06)	12	3	0	87	0.969
11	Crisis/coping plan	3.20 (0.94)	8	7	0	80	0.962
12	Seminars/meetings for relatives	1.67 (1.23)	13	1	1	100	1.000
13	Family involvement in discharge report	2.00 (1.20)	12	3	0	80	0.910
	Subscale total	2.69 (0.55)	14	1	0	–	0.979
	Scale total	2.33 (0.47)	15	0	0	–	0.991

Overall, the units routinely identified the patients’ next of kin and discussed family involvement with most of the patients (items 5 and 7). None of the units had routines to provide written information to patients and relatives about useful websites, support groups and resources, and only one site routinely provided written information about their unit’s family involvement (item 2). Five units offered information meetings/ psychoeducative seminars for relatives, but the recruitment strategies to, and attendance of, these courses varied between the units (item 12).

There was a large variation in practices between the units when it came to inviting patients and relatives to a conversation with personnel, together and/or separately, to discuss family involvement (items 6, 8, 9 and 10). Only two of the units used checklists to standardise the content of such conversations, and the topics usually covered varied between clinicians and between sites (items 3 and 4). The use of crisis/coping plans (item 11) and documentation of family involvement in the patients’ discharge report (item 13) also varied considerably.

There were small differences in average scores on several items, between the units who offered FPE and those who did not. To investigate any correlation between the BFIS scores and the units’ FPE status, we employed an independent samples Mann-Whitney U Test with two-tailed significance level α = 0.05. For the average BFIS scores we calculated U = 27.5 and *p* = 0.955. *P*-values for individual items varied greatly, from *p* = 1.0 (items 1, 3, 5 and 14) to *p* = 0.054 (item 13). Thus, no statistically significant correlation was found.

### Family psychoeducation

Eight of fifteen sites offered FPE to patients with psychotic disorders and their relatives. The percentage of patients with psychotic disorders who had received or were receiving FPE in all units was 4.2%, ranging from 0 to 17.5% between sites. In the sites that offered FPE, the percentage was 9.4%, ranging from 1.9 to 17.5%. One unit offered both FPE and another family intervention inspired by Open Dialogue [39], but the remaining seven did not provide such interventions to their patients at all.

Item distributions for the FPE and GOI scales are listed in Tables 4 and 5. The mean fidelity score on the FPE scale was 2.78, ranging from 1.00 to 4.77. However, the distribution was markedly bimodal, since the seven units who did not offer FPE were scored 1 on all items. In the eight sites that did offer FPE, the mean score was 4.34, ranging from 4.00 to 4.77, showing that all of them practiced the model with adequate fidelity. Only four sites had appointed personnel to coordinate FPE activities (item 1). In general, clinicians remained true to the structure and content of the model (items 2–6, 8, 9 and 11–13), but the use of multimedia sources varied (item 10). Active recruitment of patients and relatives to FPE was generally low, with an average fidelity score of 2.5 in the sites that offered FPE (item 14).

**Table 4:** Item distributions for the Family Psychoeducation fidelity assessment (FPE) scale

		All units (*n* = 15)				Units with FPE (*n* = 8)			
		Mean (SD)	FPE item ratings by site			Mean (SD)	FPE item ratings by site		
Item	Description		Low	Adequate	Full		Low	Adequate	Full
1	Family intervention coordinator	1.60 (1.18)	14	0	1	2.13 (1.46)	7	0	1
2	Session frequency	3.00 (1.96)	7	2	6	4.75 (0.46)	0	2	6
3	Long-term FPE	3.13 (2.07)	7	0	8	5.00 (0.00)	0	0	8
4	Quality of clinician-family alliance	2.93 (1.91)	7	3	5	4.63 (0.52)	0	3	5
5	Detailed family reaction	3.13 (2.07)	7	0	8	5.00 (0.00)	0	0	8
6	Precipitating factors	3.13 (2.07)	7	0	8	5.00 (0.00)	0	0	8
7	Prodromal signs (not rated)	–	–	–	–	–	–	–	–
8	Coping strategies	3.13 (2.07)	7	0	8	5.00 (0.00)	0	0	8
9	Educational curriculum	2.93 (1.94)	8	1	6	4.63 (0.74)	1	1	6
10	Multimedia education	2.13 (1.64)	12	0	3	3.13 (1.73)	5	0	3
11	Structured group sessions	3.13 (2.07)	7	0	8	5.00 (0.00)	0	0	8
12	Structured problem solving	3.13 (2.07)	7	0	8	5.00 (0.00)	0	0	8
13	Stage-wise provision of services	2.93 (1.94)	8	1	6	4.63 (0.74)	1	1	6
14	Assertive engagement and outreach	1.80 (0.86)	15	0	0	2.50 (0.54)	8	0	0
	Scale total	2.78 (1.74)	7	8	0	4.34 (0.30)	0	8	0

**Table 5:** Item distributions for the General Organizational Index (GOI) scale

		All units (*n* = 15)				Units with FPE (*n* = 8)			
		Mean (SD)	GOI item ratings by site			Mean (SD)	GOI item ratings by site		
Item	Description		Low	Adequate	Full		Low	Adequate	Full
	*Individualisation*								
2	Eligibility/client identification	1.27 (1.03)	14	0	1	1.50 (1.41)	7	0	1
4	Assessment	2.53 (1.69)	10	2	3	3.87 (1.13)	3	2	3
5	Individualised treatment plan	1.73 (0.88)	15	0	0	2.38 (0.74)	8	0	0
6	Individualised treatment	2.93 (2.02)	8	0	7	4.63 (1.06)	1	0	7
12	Client choice regarding services	2.87 (1.85)	7	4	4	4.5 (0.54)	0	4	4
	*Quality improvement*								
7	Training	1.53 (1.41)	13	0	2	2.00 (1.85)	6	0	2
8	Supervision	1.87 (0.99)	14	1	0	2.62 (0.74)	7	1	0
9	Process monitoring	1.00 (0.00)	15	0	0	1.00 (0.00)	8	0	0
10	Outcome monitoring	1.00 (0.00)	15	0	0	1.00 (0.00)	8	0	0
11	Quality assurance	1.00 (0.00)	15	0	0	1.00 (0.00)	8	0	0
	*Additional items*								
1	Program philosophy	2.60 (1.64)	9	4	2	4.0 (0.76)	2	4	2
3	Penetration	1.00 (0.00)	15	0	0	1.00 (0.00)	8	0	0
	Scale total	1.78 (0.81)	15	0	0	2.46 (0.42)	8	0	0

A similar tendency was seen in the GOI scores, where only one unit had a standardised form of eligibility identification (item 2) and none of them had provided FPE to more than 20% of eligible patients (item 3). Our premise when rating item 3 was that all patients with psychotic disorders were eligible to receive FPE, which is probably an overestimate. The average GOI score in all 15 sites was 1.78, ranging from 1.00 to 3.00. Among the eight sites who had implemented FPE, the average GOI score was 2.46, ranging from 1.92 to 3.00, indicating that none of these had achieved an adequate integration of FPE in their organisation.

### Psychometric properties

From the present survey in 15 sites, we have calculated the percentage of exact agreement and the intra-cluster correlation coefficient (ICC) for each item, and the mean total fidelity of the BFIS scale (Table 3). These preliminary measures of IRR indicate a high level of agreement between raters, with an ICC of 0.99 for mean total fidelity.

Concerning the FPE scale, we calculated an ICC of 0.93 for mean total fidelity, whereas the GOI scale had an ICC of 0.96. Both numbers suggest a high level of agreement between raters. These calculations were only based on the results from the eight sites that offered FPE, because including the unanimous scores from the units who did not offer FPE would produce an artificially high correlation.

## Discussion

### Basic family involvement and support

The results from this study demonstrate a general lack of structures and standard procedures in Norwegian CMHCs, when it comes to family involvement and support for persons with psychotic disorders.

Several units had local resource persons with special competence in family involvement, who worked hard to increase the awareness and recognition of their field. During this survey, the project group took note of many exemplary practices that could inspire other units and clinicians in the subsequent phases of the IFIP trial. Some of the clinical sites had established local structures and routines for basic family involvement and support, and several had information meetings or other support measures for relatives.

In most units however, contact with and involvement of relatives appeared both random and inadequate, depending highly on the practice of the patient’s clinician. As such, the results of this systematic survey of mental health services is consistent with the findings of previous research on relatives’ experiences [2, 3]. The poor organisation of family involvement and support for adult relatives contrasted distinctly with the legally mandated structures, procedures and responsibilities for children as next of kin. Nearly all the units in our survey had personnel responsible for taking care of children as next of kin and written procedures on this subject, which were widely used among the remaining personnel. The legislation concerning children as next of kin was passed in 2009, whereas the guidelines on family involvement in the health- and care services were published in 2017. The differences in implementation rates may be primarily due to legal incentives (and sanctions) being more important to administrators than following guidelines, but also related to the longer time span and family work towards children receiving more attention. In any case, it shows that improvement in CMHCs’ family work is feasible with appropriate focus, support, and incentives.

Several clinicians had frequent contact with relatives by phone, and the BFIS scale does not include the penetration rate of such calls. The low percentage of relatives who were invited to a conversation at the CMHC, with or without the patient present, indicate that such conversations are not part of the standard approach in most units. The variable use of crisis plans and infrequent documentation of family involvement in the patients’ discharge reports may disrupt the continuity of care that is vital to this patient group and their next of kin.

The fact that none of the units had annual training of their clinical personnel in family involvement is a particularly important finding, since the education of health professionals in Norway have generally given limited attention to this subject. It therefore requires substantial effort within the health services to implement family involvement as a standard approach among clinicians.

There may be several reasons why family involvement has received such little attention, in both training and implementation in Norwegian mental healthcare. The research literature suggest that poor implementation is a problem internationally, and that barriers to family involvement exist on multiple levels. On a system level, these include a lack of financial incentives and explicit prioritization from managers and politicians, organisational cultures and paradigms, attitudes of leaders and staff towards evidence-based practices in general and/or family involvement in particular, inter-professional struggles, and poor access to training and supervision [4, 17, 18]. As part of the IFIP trial, we aim to investigate barriers to and facilitators for family involvement practices on a clinical, organisational and political level in the Norwegian context, trough qualitative methods.

### The BFIS scale

The present model for basic family involvement and support is novel and has not yet been investigated scientifically as a whole. It consists of elements whose rationale varies from scientific evidence to legal frameworks and rights, as well as moral obligations. This reflects the composite nature of the guidelines that the model is based on. As such, the BFIS fidelity scale is one of the first instruments of its kind to measure the implementation of guidelines and practices that are not exclusively evidence-based. We would argue that this new application of the fidelity methodology is justified, since many practices within mental health services are based on predominantly ethical and/or legal considerations, rather than expectations of treatment effect. The scale should also be appropriate to measure basic family involvement and support for patients with other forms of severe mental illness. Perhaps, with some modifications, it may be suitable for health services towards other patient groups with chronic and severe illness.

Concerning psychometric properties, the scale shows promising IRR, appears to have relevant content and captures variability in practice, but we cannot yet establish its benchmark value. The percentage of exact agreement for each item was generally high, but the lack of standard procedures and high variability among practitioners complicated the scoring of some items. The fact that units who offered FPE did not score significantly better or worse on the BFIS scale, indicates that the scale measures practices that are independent of FPE, which may support the introduction of the scale. The Mann-Whitney U test was appropriate, because of the low sample size and the irregular distribution of the data. However, given the low sample size and generally low power of non-parametric tests, this lack of significant correlation should be interpreted with caution.

### Family psychoeducation

The Norwegian guidelines recommending structured family interventions as a first-line treatment for persons with psychotic disorders were published in 2013, and the evidence supporting such interventions has been available for much longer. Yet, only 4.2% of the patients with psychotic disorders in our participating units had received FPE, and nearly half of the sites did not offer FPE or any family intervention at all. These findings are consistent with the international research literature [4, 5].

In the units who did provide FPE, the penetration rate was low and the majority of sites lacked structures and procedures to identify and recruit eligible patients, and to coordinate FPE activities. However, the quality of the FPE provided was consistently high, suggesting that the training and supervision the units had received from The Early Intervention in Psychosis Advisory Unit for South East Norway (TIPS Sør-Øst) was excellent. Yet, training and guidance in FPE by itself did not appear sufficient to implement the intervention as an integrated part of the unit’s organisation and practice. This is revealed by the poor GOI scores, which illustrate the benefits of using scales that not only measure practice and content, but also organisation, implementation and individualisation [38]. The BFIS scale is an attempt to combine these elements in a single instrument.

### Strengths and limitations

One advantage of fidelity measurements is the standardised and structured assessment of all units in a sample [27]. A weakness of this approach is that one does not investigate practices that are not addressed by the instruments. However, in our fidelity reports we recorded if the units had any family involvement practices that our instruments failed to credit, and these were few.

We could have included additional data sources, such as observations of FPE-sessions and interviews with patients and relatives. A review of randomly chosen patient records at each site would have strengthened the validity of our survey, particularly of the penetration rate items. Unfortunately, gaining access to the patient record software proved so legally complicated that this endeavour had to be abandoned.

When it comes to the representability of the sample, we only included units from the southeast of Norway and we exclusively measured their practice towards patients with psychotic disorders and their relatives. In terms of external validity, these findings do not necessarily reflect the situation in other regions of the country and/or practice towards other patient groups. Yet, the sample of clinical units in our investigation include both urban and rural sites and serves approximately 25% percent of the Norwegian population. Consequently, our survey measures specialist health services towards a large part of this patient group and their relatives in Norway. We have little reason to believe that the clinical units’ family involvement practices towards other patient groups with severe mental illness were more systematic or of higher quality.

The recruitment of clinical units, both in terms of sample size and type of units, was made considering the trial as whole, and not specifically this cross-sectional sub-study. It could be argued that units who did not offer FPE had greater incentives to join our research project, which could lead to a form of selection bias. However, most of the CMHCs in the region agreed to participate, and the ratio of units who offered FPE versus those who did not was the same among participant and non-participant CMHCs.

## Conclusions

This cross-sectional assessment confirmed our hypothesis; that the uptake of the national guidelines on family involvement for persons with psychotic disorders in Norwegian CMHCs was generally poor. Few patients and relatives had received FPE, which is a key ingredient in the evidence-based treatment for these patients. However, the quality of FPE was consistently high, when provided. Our novel fidelity scale, which measures basic family involvement and support, shows promising preliminary psychometric properties and may prove a useful tool to improve the quality of health services. There is a need to increase the implementation and penetration rate of family involvement practices for patients with psychotic disorders and their relatives in Norway.

## Supplementary Information

**Additional file 1.** STROBE Statement—Checklist of items that should be included in reports of cross-sectional studies.

**Additional file 2.** Fidelity scale for Basic Family Involvement and Support (BFIS).

## Data Availability

The datasets used and/or analysed during the current study are available from the corresponding author on reasonable request.

## Abbreviations

AOT: Assertive outreach team
BFIS: Basic family involvement and support
CMHC: Community mental health centre
CTO: Community treatment order
DDT: Dual diagnosis team
FPE: Family psychoeducation
FTE: Full-time equivalent staff
GOI: General organizational index
GPC: General psychiatric clinic
ICC: Intra-class correlation coefficient
IFIP: Implementation of family involvement for persons with psychotic disorders
IRR: Interrater reliability
POC: Psychosis outpatient clinic
STROBE: Strengthening the reporting of observational studies in epidemiology
