# Supplementary material for: Family involvement practices for persons with psychotic disorders in community mental health centres – a cross-sectional fidelity-based study
Source: BMC Psychiatry. 2021 Jun 2;21:285. doi: 10.1186/s12888-021-03300-4 (PMC8170939; doi:10.1186/s12888-021-03300-4)
Supplement: Supplementary file 2 — Additional file 2. Fidelity scale for Basic Family Involvement and Support (BFIS). [file 12888_2021_3300_MOESM2_ESM.pdf]

	Fidelity scale for Basic Family Involvement and Support (BFIS)					
	**Item**	**Score:**	**1**	**2**	**3**	**4**	**5**

**1**	**Training and supervision of health personnel**<br>The unit shall ensure that annual training in basic family involvement and support is offered to all health personnel at the unit. The training should cover the following subjects:<br><br>a) The importance of family involvement and the benefits of following the national guidelines.<br><br>b) How to approach relatives in a good way, and show acknowledgment through small gestures (offer coffee/water, wish them welcome etc.).<br><br>c) The legal rights and roles of patients and relatives, and the health services' obligations towards them.<br><br>d) How to promote family- and patient involvement, good communication and cooperation with relative and patient in different phases and situations during the treatment of psychotic disorders.<br><br>e) Common challenges related to the carer role, how health personnel can support relatives, and about support measures in and outside the health services.<br><br>f) Professional, legal and ethical challenges that may arise during family involvement and strategies to handle these.	0-1 criterion	2-3 criteria	4-5 criteria	6 criteria	7 criteria

	Access to supervision<br>g) Health personnel at the unit shall have access to supervision in family involvement (e.g. from a family coordinator, personnel with training in family psychoeducation, reflection groups or a clinical ethics committee.)					
**2**	**Family coordinator - General structure and responsibilities:**<br><br>a) One or more of the unit's health personnel is/are designated specifically to coordinate basic family involvement and support at the unit. A proportion of the working hours are allocated to this task. (This item counts as 2 criteria if there is allocated time to the task, or 1 criterion if a coordinator is merely appointed).<br><br>b) The coordinator receives training in the role, and has access to supervision and exchange of experience annually.<br><br>c) The coordinator receives training in family psychoeducation (FPE), and has access to supervision and exchange of experience in FPE annually.	0-1 criterion	2-3 criteria	4-5 criteria	6-7 criteria	8 criteria

	d) The coordinator should have good knowledge of, and a written updated overview of, support measures in and outside the health services.<br><br>e) The coordinator should have good knowledge of, and a written overview of, important barriers to family involvement and possible strategies to handle them.<br><br>f) Written information about the unit's family involvement (how and why) is available and handed out routinely to patients and relatives. (The criterion is met as long as somebody at the unit takes care of this).<br><br>g) Written information about useful web resources and support groups is available and handed out routinely to patients and relatives. (The criterion is met as long as somebody in the unit takes care of this).					
3	**Conversation(s) with the patient without the relative(s) present.**<br><br>Can be performed by the family coordinator or other health personnel.<br><br>a) Patients at the unit get at least one consultation/conversation where family involvement is the main topic. (Counts as 2 criteria).	0-1 criterion	2-3 criteria	4-5 criteria	6-7 criteria	8 criteria

	b) A written guide/ checklist of items that should be covered is used.<br><br>Subjects concerning family involvement that should be covered in one or more conversations with the patient alone:<br><br>c) Ask the patient: "What is important for you to talk about regarding family involvement?"<br><br>d) Ask how the patient experience the relationship to his/her relatives, including any children.<br><br>e) Investigate whether the patient is exposed to violence and/or abuse from his/her relatives.<br><br>f) Talk with the patient about family involvement, confidentiality, and conflicts of interest, and elicit the patient's preferences and concerns.<br><br>g) Include issues concerning young children, their needs, and parental responsibility, (if the patient has children).					

	Fidelity scale for Basic Family Involvement and Support (BFIS)					
	**Item** **Score:**	**1**	**2**	**3**	**4**	**5**
**4**	**Conversation(s) with the relative(s) without the patient present.**<br><br>Can be performed by the family coordinator or other health personnel.<br><br>a) Relatives are invited to a separate conversation, to talk about family involvement and other relevant subjects. (Counts as 2 criteria).<br><br>b) A written guide/ checklist of items that should be covered is used.<br><br>Subjects concerning family involvement that should be covered in one or more conversations with the relative(s) alone:<br><br>c) Ask the relative(s): "What is important for you to talk about?"<br><br>d) Talk with the relative(s) about relevant roles, responsibilities, and legal regulations, e.g. related to family involvement, confidentiality and documentation.<br><br>e) Ask how the relative(s) experience the relationship to the patient. Listen to the relatives' concerns and elicit what they already know about the patient.	0-1 criterion	2-4 criteria	5-6 criteria	7-8 criteria	9-10 criteria

	Fidelity scale for Basic Family Involvement and Support (BFIS)					
	**Item** **Score:**	**1**	**2**	**3**	**4**	**5**
	f) Identify the relatives' tasks, resources, and carer burdens to be able to assess their need for support, and advise them on where they may get it.<br><br>g) Talk with the relative(s) about common economic, social and health-related challenges related to the carer role, and about strategies to handle them and where one can get further support if necessary.<br><br>h) If children are affected, talk with the relatives about the parental role and responsibilities, what information and follow-up the children need and have received, and give advice about where one can get help to meet the children's needs.<br><br>i) Investigate whether the relative(s) is/are exposed to violence and/or abuse from the patient.					

	Fidelity scale for Basic Family Involvement and Support (BFIS)					
	**Item** **Score:**	**1**	**2**	**3**	**4**	**5**
	**Item 5-13:**<br>**Clinical practice towards patients and relatives, measured by penetration rate. (Can be taken care of by the family coordinator and/or other health personnel).**					
5	Ensure that personnel identify and document who are the patient's relatives (the next of kin, other central persons and additional network). The percentage of patients for whom this is done.	0-19%	20-39%	40-59%	60-79%	80-100%
6	Patients at the unit get at least one consultation/ conversation where family involvement is the main topic. The percentage of patients for whom this is done.	0-19%	20-39%	40-59%	60-79%	80-100%
7	Ensure that the subject family involvement is discussed in one or more conversations. The percentage of patients for whom this is done.	0-19%	20-39%	40-59%	60-79%	80-100%
8	Ensure that the relative(s) is/are invited to at least one conversation, without the patient present, to discuss family involvement, family psychoeducation, and other relevant subjects. The percentage of patients whose relatives have been invited to at least one such conversation.	0-19%	20-39%	40-59%	60-79%	80-100%
9	Ensure that patients and their relative(s) are invited to at least one joint conversation, preferably as a part of family psychoeducation, to share what can be shared and sum up. The percentage of patients (with their relatives) that have been invited to at least one such conversation.	0-19%	20-39%	40-59%	60-79%	80-100%

	Fidelity scale for Basic Family Involvement and Support (BFIS)					
	**Item** **Score:**	**1**	**2**	**3**	**4**	**5**
10	The patient's primary clinician attends at least one joint conversation with patient and relative(s). The percentage of patients that have been invited to at least one such conversation with their relative(s) and primary clinician.	0-19%	20-39%	40-59%	60-79%	80-100%
11	A crisis plan/ coping plan is developed (it should be updated annually and found easily accessible in the patient records). The percentage of patients that have an applicable crisis/coping plan.	0-19%	20-39%	40-59%	60-79%	80-100%
12	Psychoeducative seminars/ information meetings for relatives, with relevant content, are arranged at least once a year. The percentage of patients whose relatives are invited to/ informed about such a seminar.	0-19%	20-39%	40-59%	60-79%	80-100%
13	The family involvement performed is summed up in the patient's discharge report to the municipal health- and care services. (Not just who is the next of kin, but what has been accomplished and agreed, information about the crisis plan, and any advice/plan for further family involvement. It should also be documented when family involvement has not been performed and why). The percentage of patients for whom this is done.	0-19%	20-39%	40-59%	60-79%	80-100%

	Fidelity scale for Basic Family Involvement and Support (BFIS)					
	**Item** **Score:**	**1**	**2**	**3**	**4**	**5**
14	**Implementation measures**<br><br>a) An implementation team, which includes the unit leader, is established at the unit to improve family involvement practices. (The leader does not necessarily have to be part of the team, but must have regular contact with the team).<br><br>b) The implementation team has routines to get input from patients and relatives.<br><br>c) Regular evaluation of the implementation process is performed, and the results are used actively to manage improvements. (E.g. fidelity measurements or other forms of systematic monitoring/internal control).<br><br>d) Regular evaluation of how both patients and relatives experience their involvement at the unit is performed, and the results are used actively to manage improvements. (E.g. annual questionnaires or focus groups).<br><br>e) The unit and the implementation team has a written overview of common barriers to family involvement, including ethical and legal dilemmas on different levels, and possible strategies to handle them. This overview, with strategies, is available and used to support the implementation.	0-1 criterion	2 criteria	3 criteria	4 criteria	5 criteria

	Fidelity scale for Basic Family Involvement and Support (BFIS)					
	**Item Score:**	**1**	**2**	**3**	**4**	**5**
